# Genome-wide identification of chitinase genes in *Thalassiosira pseudonana* and analysis of their expression under abiotic stresses

**DOI:** 10.1186/s12870-021-02849-2

**Published:** 2021-02-10

**Authors:** Haomiao Cheng, Zhanru Shao, Chang Lu, Delin Duan

**Affiliations:** 1 Key Laboratory of Experimental Marine Biology, Center for Ocean Mega-Science, Institute of Oceanology, Chinese Academy of Sciences, Qingdao, 266071 P. R. China; 2 Laboratory for Marine Biology and Biotechnology, Pilot Qingdao National Laboratory for Marine Science and Technology, Qingdao, 266237 P. R. China; 3 University of Chinese Academy of Sciences, Beijing, 100049 P. R. China; 4 State Key Laboratory of Bioactive Seaweed Substances, Qingdao Bright Moon Seaweed Group Co Ltd, Qingdao, 266400 P. R. China

**Keywords:** Abiotic stress, Chitinase, Enzymatic activity, Gene expression, Gene family, *Thalassiosira pseudonana*

## Abstract

**Background:**

The nitrogen-containing polysaccharide chitin is the second most abundant biopolymer on earth and is found in the cell walls of diatoms, where it serves as a scaffold for biosilica deposition. Diatom chitin is an important source of carbon and nitrogen in the marine environment, but surprisingly little is known about basic chitinase metabolism in diatoms.

**Results:**

Here, we identify and fully characterize 24 chitinase genes from the model centric diatom *Thalassiosira pseudonana*. We demonstrate that their expression is broadly upregulated under abiotic stresses, despite the fact that chitinase activity itself remains unchanged, and we discuss several explanations for this result. We also examine the potential transcriptional complexity of the intron-rich *T. pseudonana* chitinase genes and provide evidence for two separate tandem duplication events during their evolution.

**Conclusions:**

Given the many applications of chitin and chitin derivatives in suture production, wound healing, drug delivery, and other processes, new insight into diatom chitin metabolism has both theoretical and practical value.

**Supplementary Information:**

The online version contains supplementary material available at 10.1186/s12870-021-02849-2.

## Background

Chitin, β-1,4-linked *N*-acetyl-D-glucosamine, is the second most abundant natural polymer on earth after cellulose. Chitin is found in insect and crustacean exoskeletons, mollusc endoskeletons, and the cell walls of fungi and diatoms [1]. Chitin and its derivative, chitosan, are used in suture production, wound healing, drug delivery, vehicles, scaffolds, and other applications [2, 3]. Chitin has three main crystal structures: α-, β-, and γ-chitin [4]. Among these, β-chitin displays a parallel polysaccharide structure that confers greater reactivity, swelling, and solubility compared with the other two chitin types [5, 6]. β-chitin is only found in certain marine organisms, including molluscs [6, 7], tubeworms [8], and diatoms [9].

Diatoms are dominant primary producers that account for 40% of primary production in the oceans and perform 20% of annual global carbon fixation [10]. Chitin is a fundamental structure within the cell walls of diatoms and serves as a supporting scaffold for biosilica deposition [11]. Flexible chitin spines have been proposed to control cell buoyancy or sinking [12], and chitin can form a network around diatom cells that promotes the adhesion of bacteria such as *Vibrio parahaemolyticus* [13]. As a nitrogenous polysaccharide, chitin is an important source of carbon and nitrogen in the marine environment. It has been suggested that these two elements would quickly disappear from the ocean if chitin stopped circulating [14, 15]. To date, chitin metabolism has been investigated in two diatom genera, *Thalassiosira* and *Cyclotella*, due to the completion of their genome sequencing [16–18].

As known β-chitin producers, centric diatoms are presumed to contain special enzymes that catalyze chitin biosynthesis and degradation. Chitinases (EC 3.2.1.14) are one of the largest groups of hydrolases and decompose chitin into *N*-acetylglucosamines. Chitinase and its derivatives have been broadly applied in agriculture, pathogenicity, allergenicity, and health [19–21]. Chitinases have been found in diverse organisms from different kingdoms of life, ranging from bacteria and higher plants to animals [22]. They are classified into glycosyl hydrolase families 18 and 19 (GH18 & GH19) based on the sequence homology of their catalytic domains [23]. GH18 chitinases are widely distributed among organisms such as viruses, bacteria, fungi, yeast, plants, and animals, whereas GH19 chitinases are found almost exclusively in plants [24]. The two chitinase families possess distinct sequence features and different three dimensional (3D-structures, suggesting that they have descended from a different ancestor [25]. Liu et al. (2018) described the sequence homology, domain architectures, and gene expression patterns of chitinases in the oriental fruit fly and proposed their putative physiological functions [26]. Chen et al. (2018) characterized the chitinase gene family in *Brassica rapa* and examined its role in clubroot resistance [27]. Bartholomew et al. (2019) identified cucumber chitinase genes and characterized their evolution and expression responses to *Fusarium oxysporum* [28]*.* Cao & Tan (2019) studied the tomato chitinase gene family and its response to *Sclerotinia sclerotiorum* and abiotic stresses including low temperature, high temperature, drought, and salt [29]. Previous work has focused on chitinase functions in nutrition, morphogenesis, and defence against pathogens and abiotic stresses in bacteria, fungi, insects, plants, animals, and other organisms [30], but few studies have examined chitinases in diatoms.

Here, we investigated the chitinase gene family in the model *Thalassiosira* species *Thalassiosira pseudonana*, making use of its completely sequenced genome [16]. We identified 24 chitinase genes and analyzed their sequence structural features, scaffold locations, phylogenetic relationships, stress-related *cis-*acting elements, subcellular locations, and responses to abiotic stresses at the transcriptional and enzymatic levels. This is the first study to investigate the characteristics of chitinase family members in diatoms. Our results provide insight into β-chitin degradation in diatoms and can support in vitro applications of chitinases derived from diatoms.

## Results

### Genome-wide identification and analysis of chitinase genes in *T. pseudonana*

A total of 24 non-redundant chitinase genes were identified in the genome of *T. pseudonana* and designated *TpChi1–24* based on their chromosomal locations. Gene names, gene IDs, chromosomal locations, exon numbers, amino acid sequence lengths, molecular weights (MW), and theoretical isoelectric points (pIs) are presented in Table 1. The sequence lengths of the chitinases ranged from 417 (TpChi1) to 3512 (TpChi6) amino acid (aa) residues, with an average length of 1517 aa. The relative molecular mass varied from 46.4 kDa (TpChi1) to 373.1 kDa (TpChi6), and the pI values ranged from 4.09 (TpChi6) to 5.42 (TpChi16).

**Table 1:** Features of the identified *T. pseudonana* chitinase genes

Name	JGI gene ID	Scaffold	Genomic location	Exon	AA	MW(KDa)	pI
*TpChi1*	21,369	2	chr_2:1,497,142-1,498,555 (+)	2	417	46.40	4.86
*TpChi2*	21,501	2	chr_2:2,082,322-2,089,884 (−)	16	2070	232.39	5.13
*TpChi3*	21,626	3	chr_3:306,112–310,877 (−)	4	1464	159.27	4.64
*TpChi4*	21,917	3	chr_3:1,759,345-1,764,662 (−)	7	1498	165.71	5.21
*TpChi5*	22,088	4	chr_4:199,894–204,931 (−)	17	1087	118.17	4.51
*TpChi6*	22,238	4	chr_4:960,301–971,814 (+)	8	3512	373.14	4.09
*TpChi7*	22,376	4	chr_4:1,635,653-1,638,316 (+)	5	684	74.97	4.66
*TpChi8*	5406	5	chr_5:1,117,857-1,121,711 (+)	14	901	96.61	4.75
*TpChi9*	22,909	5	chr_5:2,040,965-2,049,241 (+)	9	2494	273.21	4.44
*TpChi10*	22,915	5	chr_5:2,100,817-2,105,674 (−)	13	785	87.47	4.61
*TpChi11*	6636	7	chr_7:81,934–86,292 (+)	5	1349	146.67	4.47
*TpChi12*	23,384	7	chr_7:448,791–455,002 (−)	8	1819	196.99	4.49
*TpChi13*	23,470	7	chr_7:953,351–956,745 (+)	3	1031	109.60	4.28
*TpChi14*	23,629	7	chr_7:1,845,016-1,855,061 (−)	15	2773	304.08	4.31
*TpChi15*	23,700	8	chr_8:274,001–278,110 (−)	7	1116	119.55	4.44
*TpChi16*	8279	10	chr_10:177,498–181,104 (+)	10	911	101.63	5.42
*TpChi17*	24,326	11a	chr_11a:339,905–344,411 (−)	7	1113	118.52	5.08
*TpChi18*	24,421	12	chr_12:128,580–139,130 (−)	6	3325	359.94	4.25
*TpChi19*	11,110	19a_19	chr_19a_19:163,538–171,325 (−)	8	2241	241.48	4.37
*TpChi20*	11,125	19a_19	chr_19a_19:210,121–212,567 (−)	8	656	71.64	4.46
*TpChi21*	11,236	19a_19	chr_19a_19:528,078–531,770 (+)	4	1122	121.35	4.59
*TpChi22*	11,237	19a_19	chr_19a_19:532,139–535,390 (+)	8	877	94.94	4.35
*TpChi23*	11,963	22	chr_22:1,024,768-1,030,124 (−)	13	1438	156.88	4.31
*TpChi24*	25,849	23	chr_23:244567–250,756 (+)	9	1735	187.00	4.66

Table S1 lists predicted signal peptides, transmembrane helices (TMHs), and subcellular localizations for the chitinases. HECTAR predictions indicated that up to half of them (12 out of 24) possessed signal peptides. Nine (TpChi9, TpChi13, TpChi14, TpChi17, TpChi18, TpChi20, TpChi21, TpChi22, and TpChi23) were predicted to contain signal peptides by all three programs (SignalP-5.0, HECTAR, and ASAFIND), suggesting that these chitinases are involved in the secretory pathway. Twelve (TpChi1, TpChi2, TpChi3, TpChi4, TpChi5, TpChi7, TpChi8, TpChi10, TpChi11, TpChi15, TpChi16, and TpChi19) were predicted by HECTAR to have type П signal anchors, indicating that they may be Type П transmembrane proteins with an N-in orientation and maybe localized on chloroplast or mitochondrial membranes. Six (TpChi2, TpChi5, TpChi9, TpChi14, TpChi22, TpChi23) were predicted to contain TMHs, suggesting a subcellular localization at the plasma membrane or the endomembrane system.

### Gene structures, motifs, and conserved domains of the *TpChi*s genes

**Fig. 1:**
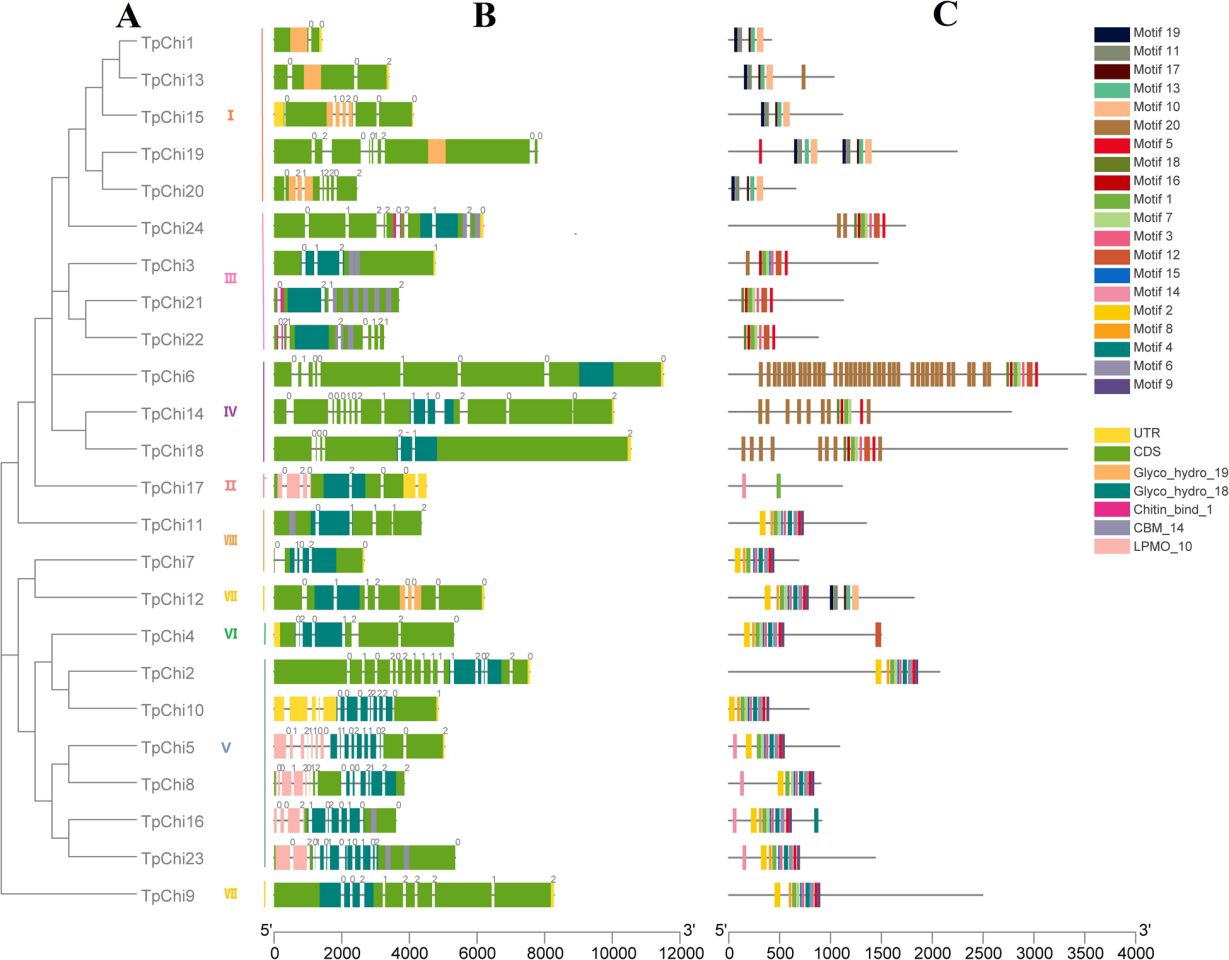
Phylogenetic relationships, gene structures, and analysis of domains and conserved motifs of the *T. pseudonana* chitinases. **a** Phylogenetic tree of 24 *T. pseudonana* chitinase proteins. **b** Gene exon/intron organization and domain distribution of the *T. pseudonana* chitinases. Grey lines represent introns, and the numbers 0, 1, and 2 represent their splicing phases. Yellow boxes represent untranslated regions (UTRs), and green boxes represent coding sequences (CDSs). Five chitinase-related domains, Glyco_hydro_19, Glyco_hydro_18, Chitin_bind_1, CBM_14, and LPMO_10, are indicated with orange, blue-green, magenta, grey, and salmon boxes, respectively, in the CDS translated regions. The lengths of exons and introns can be inferred by the scale at the bottom. **c** Distribution of conserved motifs in the *T. pseudonana* chitinases. Twenty putative motifs are indicated with different coloured boxes. Table S2 presents details of the motifs

All chitinase genes contained at least two exons and one intron, and *TpChi5* contained 17 exons, the most of all the chitinase genes. One quarter of the *TpChi*s had more than ten introns (6, 25%), over one half had seven or more introns (14, 58%), and only *TpChi1* had a single intron (Fig. 1).

The sequence differences within the chitinase family were further analyzed by examining their conserved domains and motifs using the Pfam database and the MEME program, respectively. Twenty conserved motifs were identified in the *T. pseudonana* chitinase proteins. Their lengths varied from 15 to 65 aa, and their details are presented in Table S2. The Glyco_hydro_18 domain was identified in nineteen chitinases and the Glyco_hydro_19 domain in six. Notably, chitinase TpChi12 was found to possess both Glyco_hydro_18 and Glyco_hydro_19 domains. Three other auxiliary domains, Chitin_bind_1 (PF00187), CBM_14 (Peritrophin A, PF01607), and LPMO_10 (PF03067), were detected in 10 protein sequences (41.7%). In addition, three motif combinations corresponding to the two Glyco_hydro domains were identified. The combination of motifs 19, 11, 17, 13, and 10 was detected in the Glyco_hydro_19 domain, which was identified in chitinases TpChi1, TpChi12, TpChi13, TpChi15, TpChi19, and TpChi20. Two other highly conserved motif combinations were identified in the Glyco_hydro_18 domain. Chitinases TpChi3, TpChi6, TpChi14, TpChi18, TpChi21, TpChi22, and TpChi24 contained the combination of motifs 18, 16, 1, 7, 3, 12 and 5, whereas TpChi2, TpChi4, TpChi5, TpChi7, TpChi8, TpChi9, TpChi10, TpChi11, TpChi12, TpChi16, and TpChi23 contained the combination of motifs 2, 8, 1, 7, 15, 3, 4, 6, 3, and 9. TpChi17 was an exception, containing only motifs 1 and 14. In general, chitinases in the same group had similar motif compositions (Fig. 1).

### Phylogenetic analysis of representative *Thalassiosira* chitinases

**Fig. 2:**
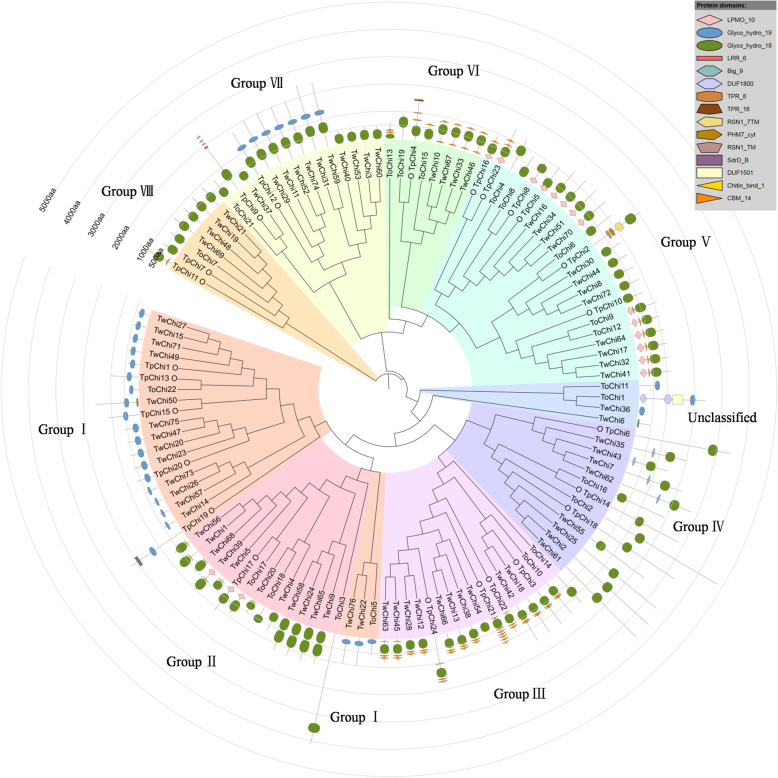
Phylogenetic relationships and domain architectures of the 122 chitinases (Chis) from *Thalassiosira oceanica* (To), *Thalassiosira pseudonana* (Tp), and *Thalassiosira weissflogii* (Tw). The *T. pseudonana* chitinases are indicated using the symbol “O”. Inner layer: An unrooted phylogenetic tree constructed with MEGA using the NJ (neighbor-joining) method and 1000 bootstrap replicates. The eight groups (I–VII) are distinguished by colours, and the four unclassified chitinases are shown in a light blue shade. Outer layer: Distribution of the chitinase protein domains, denoted by boxes of different colours and shapes. The glyco_hydro_19 domains are represented by blue oval boxes, and the glyco_hydro_18 domains are represented by green oval boxes; their positions can be inferred by the circular scales around the tree

An unrooted phylogenetic tree was constructed to reveal the evolutionary relationships among the chitinases of *T. pseudonana*, *T. oceanica* (22, Table S3) and *T. weissflogii* (76, Table S4). A total of 122 protein sequences from the three *Thalassiosira* species were used to build the phylogenetic tree (Fig. 2). The *T. pseudonana* chitinases were classified into eight groups (I–VII) based on the topology of the phylogenetic tree and the domain architectures of the protein sequences. GroupV was the largest and contained 6 members from *T. pseudonana*, 5 from *T. oceanica*, and 12 from *T. weissflogii*. A total of 15 domains were detected in the 122 chitinases from all three *Thalassiosira* species (Fig. 2). Groups II–VII all contained chitinases with the Glyco_hydro_18 catalytic domain, and the chitinases in GroupI (except for TwChi50) contained only the Glyco_hydro_19 domain. Some chitinases in GroupII (TwChi9, TwChi24, TwChi58, and TwChi65) contained two Glyco_hydro_18 domains. Interestingly, seven chitinases (TpChi12, TwChi11, TwChi29, TwChi31, TwChi52, TwChi59, and TwChi74) in GroupVII and TwChi50 in GroupI contained both the Glyco_hydro_18 and the Glyco_hydro_19 catalytic domains. Overall, chitinases in the same group had similar domain architectures.

### Scaffold locations and gene duplication of the *TpChi* genes

**Fig. 3:**
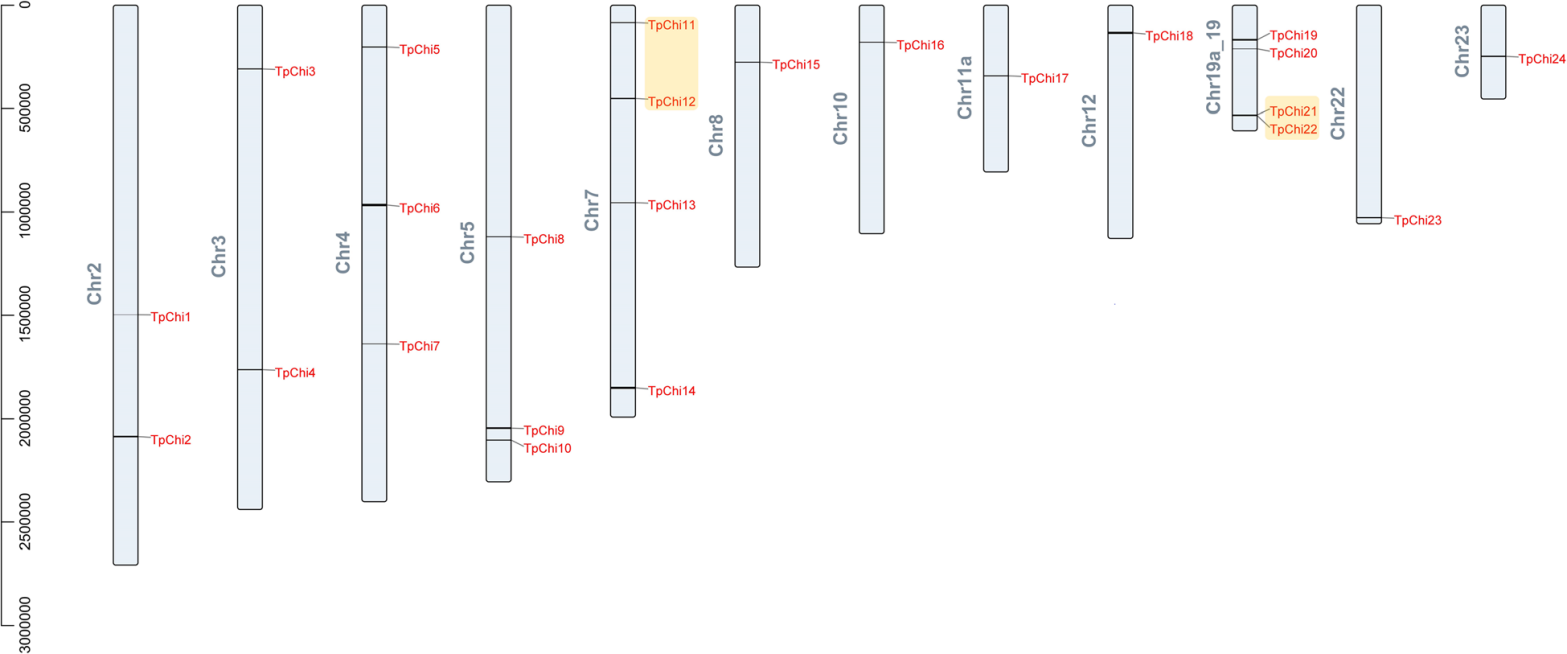
Scaffold locations and gene duplications of the *TpChi*s. The tandem duplicated gene pairs are marked by yellow rounded rectangles

The 24 *TpChi*s were assigned to 12 scaffolds (Fig. 3 and Table 1), and the distribution of chitinase genes on each scaffold was uneven. Chr7 and Chr19a_19 contained the largest number of chitinase genes (4), whereas only a single chitinase gene was located on Chr8, Chr10, Chr11a, Chr12, Chr22, and Chr23. No segmental duplication were observed among the chitinase gene family members. However, there were two tandemly duplicated gene pairs: *TpChi11* and *TpChi12*, and *TpChi21* and *TpChi22*. These were located on Chr7 and Chr19a_19, respectively, and accounted for 16.7% of the chitinase genes. No collinearity among chitinase gene family members was observed with MCScanX.

### Stress-related *cis*-acting elements in the *T. pseudonana* chitinase gene promoters

**Fig. 4:**
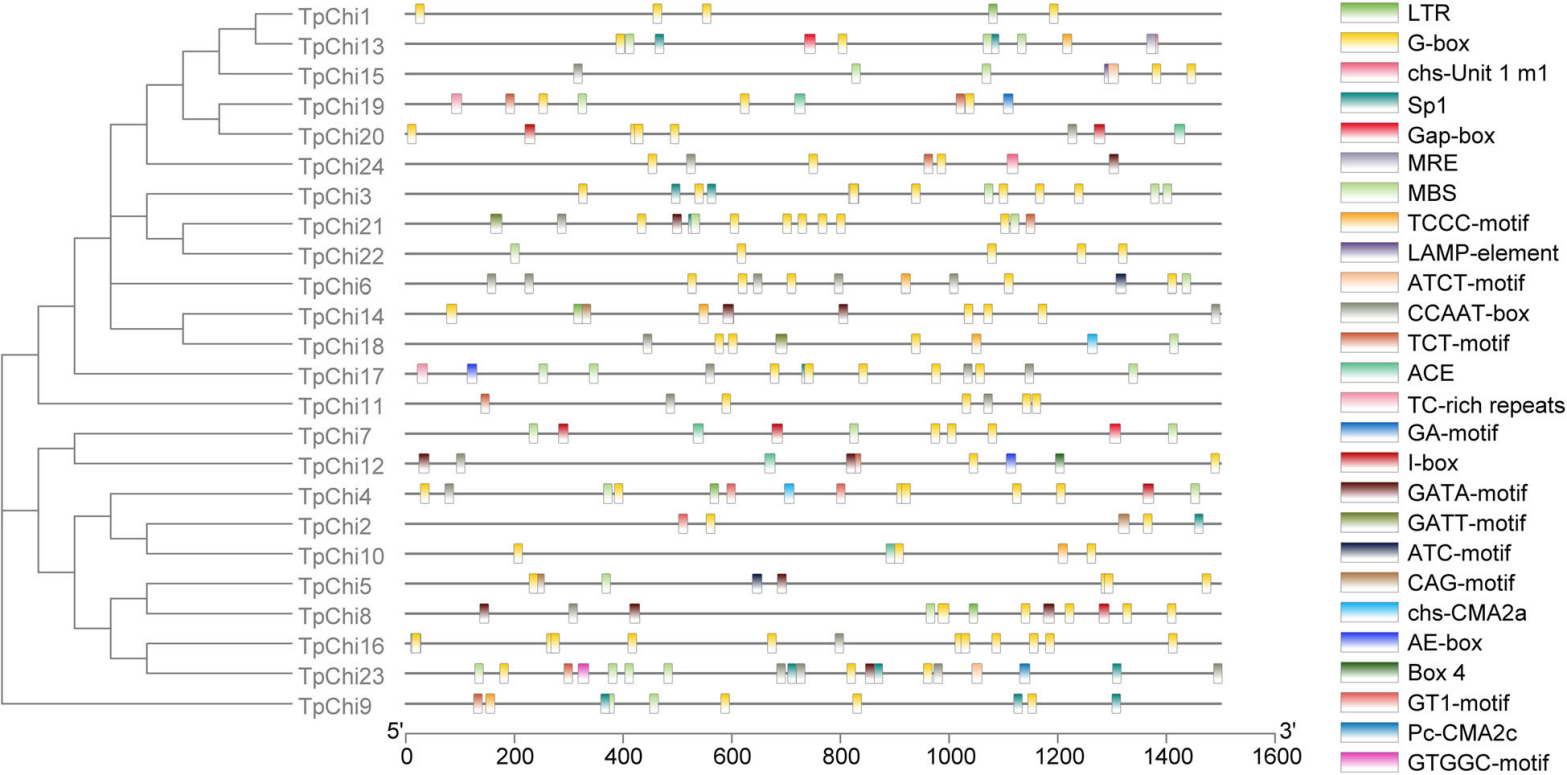
Predicted stress-related *cis-*acting elements in the promoter regions of the *TpChi*s

To investigate possible regulation mechanisms of the *TpChi*s under abiotic or biotic stresses, 1.5 kb upstream sequences (promoter regions) were analyzed using the PlantCARE database to identify *cis-*regulatory elements. Twenty-six types of 259 stress-related *cis-*acting elements were identified, including light response-related (G-box and 21 other types), low-temperature responsiveness-related (LTR), drought-inducibility-involved (MBS), and two other *cis-*acting elements involved in defence and stress responsiveness (i.e., the CCAAT-box and TC-rich repeats) (Fig. 4). All the chitinase genes contained 2–9 stress-related *cis-*acting elements in their promoter regions. The G-box was found in all *TpChi* promoters and accounted for almost half of the identified elements (122). In total, 22 types of 199 light-responsive *cis-*acting elements accounted for 76.8% of the stress-related *cis-*acting elements.

### Secondary and 3D structures of the *T. pseudonana* chitinases

**Fig. 5:**
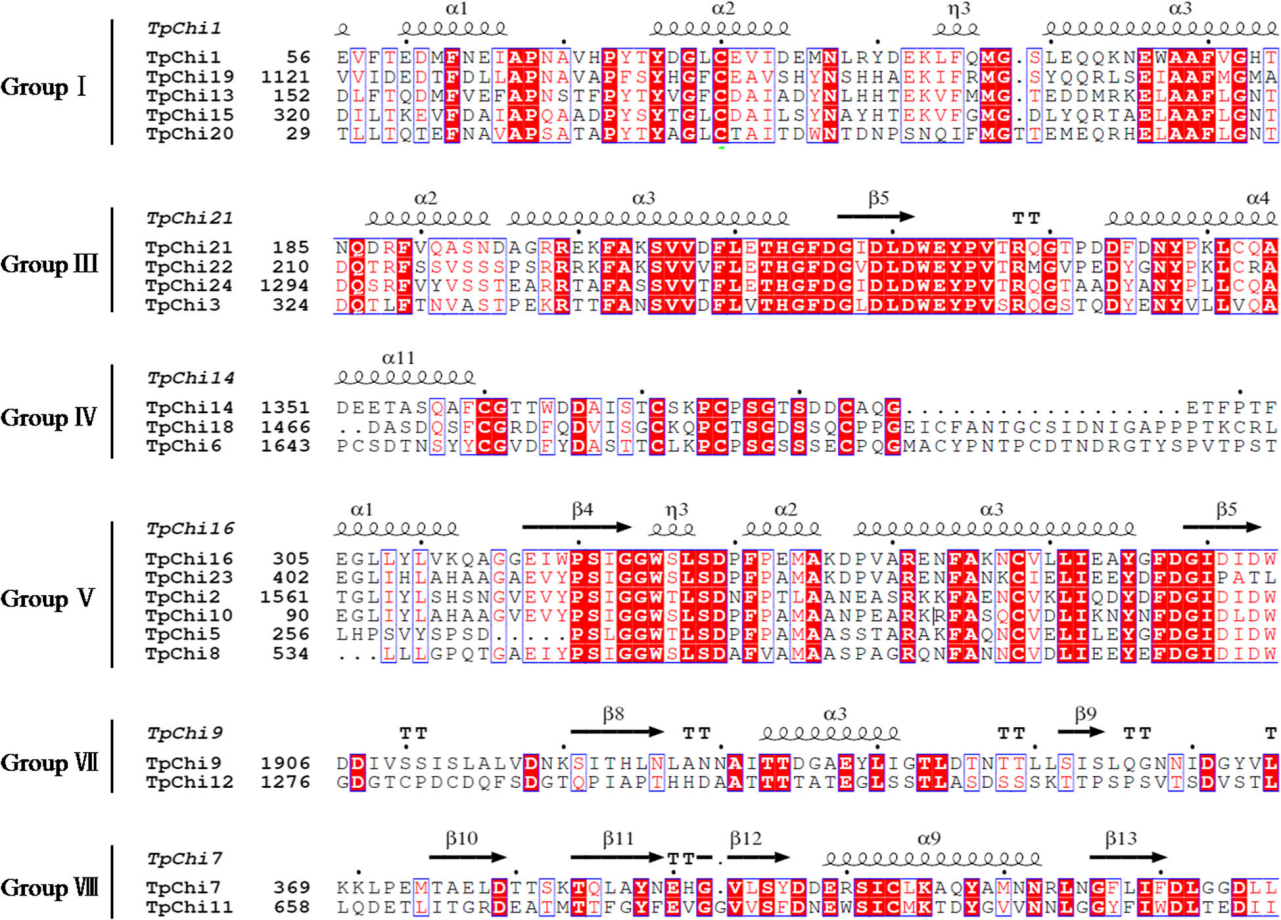
Representative secondary structures and sequence alignments of the *T. pseudonana* chitinases in each group. Amino acids in white on a red background are conserved sites, and similar amino acids are shown in red with blue rectangles. The secondary structures of the *T. pseudonana* chitinases are shown above the alignment. Alpha-helices are represented with helices symbols, β-strands with arrows, and turns with TT letters

Four secondary structures (α-helix, extended strand, β-turn, and random coil) were found in the *T. pseudonana* chitinases (Table S5). The α-helix and random coil were the dominant components of chitinase secondary structure and accounted for 24.79 and 47.46% on average. Extended strand constituted 19.63% of the secondary structures and β-turn constituted 8.12%. Figure 5 shows representative secondary structures for each group. 3D molecular modelling provides dynamic information that is usually difficult to acquire from experiments, and 3D protein models were constructed for the *T. pseudonana* chitinases to better understand their structural properties (Table S6). Most top-hit models (75%) were chitin-degrading enzymes from a wide range of phylogenetic origins, including viruses (TpChi5), bacteria (TpChi2, TpChi7, TpChi8, TpChi10, TpChi11, TpChi16, TpChi17), yeast (TpChi20), fungi (TpChi12, TpChi23), rice (TpChi1, TpChi19), insects (TpChi14, TpChi18), and humans (TpChi3, TpChi21, TpChi24). *T. pseudonana* chitinases in the same phylogenetic groups (Fig. 2) were modelled with similar 3D structures (Table S6).

### Expression analysis of *TpChi* genes under abiotic stresses

**Fig. 6:**
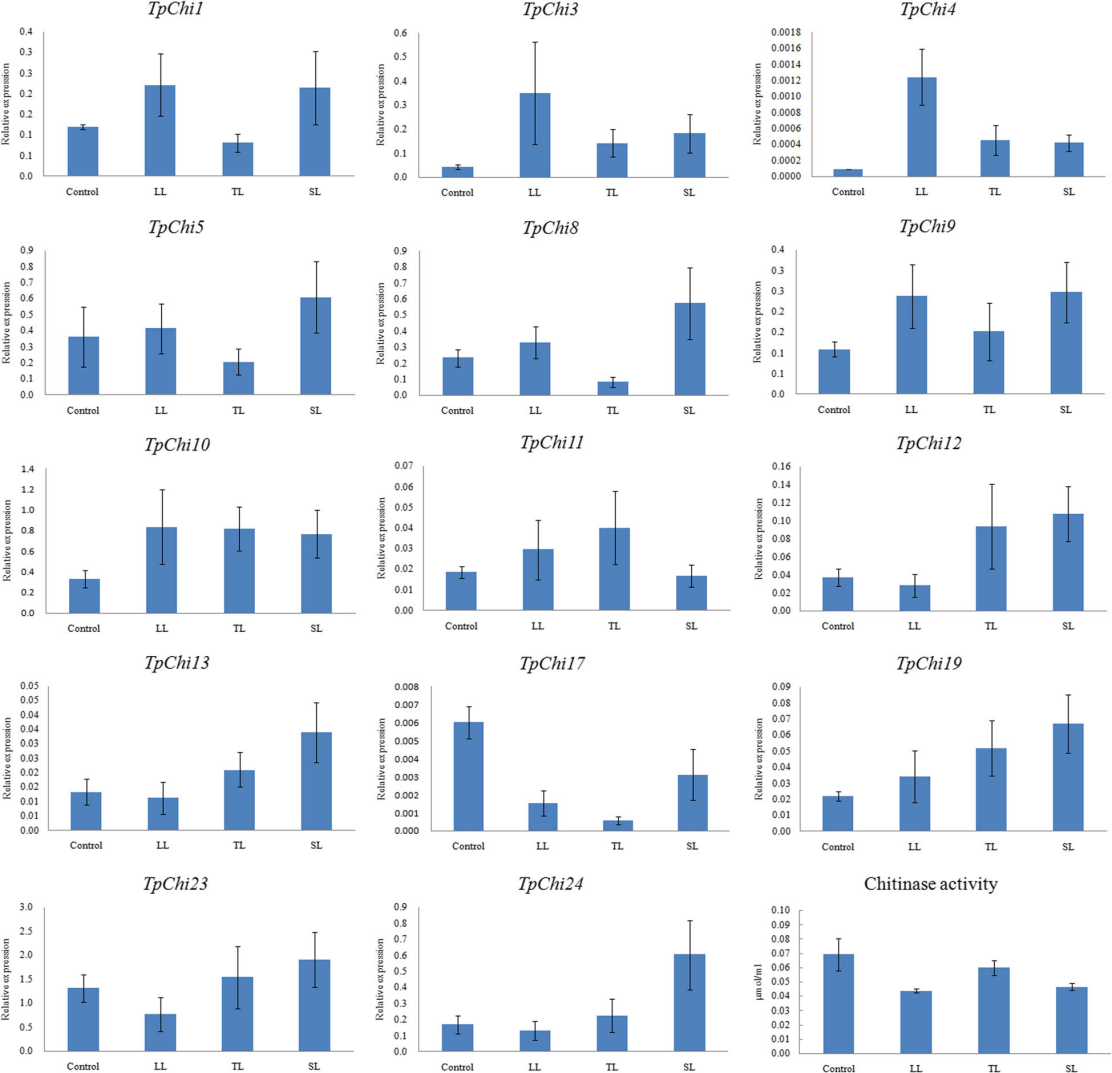
Expression profiles of 14 randomly selected chitinase genes under light-limited (LL), temperature-limited (TL), and silica-limited (SL) conditions. Data are presented as the mean ± standard deviation of three replicates. The relative transcript levels of selected genes were calculated using the 2^−ΔΔ^Ct method with beta-tubulin as the internal reference gene. Lower right: Chitinase activity of *T. pseudonana* cultures under LL, TL, and SL conditions. One-Way ANOVA statistical analysis was performed in the chitinase activity assay

To investigate individual gene responses to low irradiance (50 μmol m^− 2^ s^− 1^), low temperature (12 °C), and silica deficiency stress at the transcriptional level, 14 genes were randomly selected for quantitative real-time polymerase chain reaction (qRT–PCR) analysis from the 24 genes that were involved in abiotic stress responses according to the *cis*-acting element prediction. Generally, *TpChi*s were upregulated after 48 h of exposure to the stresses (Fig. 6). Silica depletion produced the greatest increases in transcript abundance for 12 of the 14 genes (85.7%) upregulated under silica limitation. The expression levels of 9 (64.3%) and 11 (78.6%) genes were enhanced under light-limited and temperature-limited stresses, respectively. Although most genes were upregulated under stress, the most remarkably upregulated gene was *TpChi4*, whose relative expression level increased 14-, 5-, and 5-fold under light-limited, temperature-limited, and silica-limited conditions compared with the control. By contrast, only one chitinase gene (*TpChi17*) was down regulated overall, decreasing to one-tenth of the control expression level under temperature-limited condition.

To further investigate the involvement of the chitinase gene family in abiotic stress responses at the enzymatic level, we measured chitinase activity of *T. pseudonana* under conditions of low irradiance, low temperature, and silica depletion. The activity of the *T. pseudonana* chitinases remained steady under low irradiance, low temperature, and silica deficiency (*p* > 0.05) (Fig. 6).

## Discussion

Chitin is the predominant polysaccharide in the diatom cell wall, and its various functions permit diatoms to adjust to the changeable ocean environment. Chitinase, the enzyme that degrades chitin into more valuable derivatives with shorter sugar chains and lower molecular weights, therefore deserves more attention in diatoms. Although the chitinase gene family has been widely investigated in insects, higher plants, and fungi [26, 27, 31, 32], there is little information about this gene family in Stramenopiles, and a systematic investigation has yet to be undertaken. The completeness of the *T. pseudonana* genome sequence enables the identification of putative diatom chitinase genes [16]. In the present study, *T. pseudonana* chitinase genes were identified from genome sequences, and their phylogenetic relationships within *Thalassiosira*, gene structures, conserved motifs and domains, scaffold locations, duplication events, stress-related *cis*-acting elements, and transcriptional expression profiles and enzymatic activities under abiotic stresses were investigated in an integrated manner.

### Diversity in structure and intron abundance may generate chitinases with various functions in *T. pseudonana*

In total, 24 chitinase genes were retrieved from the *T. pseudonana* genome and verified based on the identification of conserved chitinase domains. Similar numbers of chitinase genes have been reported previously in *T. pseudonana* (more than 20 chitinase-encoding genes) [33] and *Cyclotella cryptica* (22 chitinases) [17]. Members of the *T. pseudonana* chitinase family displayed different secondary and 3D structures (Tables S5, S6), suggesting that they may function in multiple biological processes, such as stress-related responses, growth, and developmental operations. Intron gain and loss amplifies the complexity of gene organization [34], and we found diverse exon-intron organization in the *T. pseudonana* chitinase genes (Fig. 1) that may have contributed to their structural diversity during evolution. Importantly, the *TpChi*s generally possessed a large number of introns. Twenty-five percent had more than 10 introns, over half had 7 or more introns (14, 58%), and members of GroupV had the most abundant introns (Fig. 1). When abundant introns are embedded in gene sequences, alternative splicing (AS) is likely to occur during gene transcription. In eukaryotes, AS spatially and temporally regulates gene expression by selectively preserving or removing some exons, thereby producing multiple mRNAs [35]. The many possible AS variants of the *TpChi*s may therefore enlarge the diversity of the *T. pseudonana* chitinase transcriptome and proteome.

### Tandem duplication and genetic diversification may co-affect the evolution of the *TpChi* gene family

*T. pseudonana* chitinase genes were classified into eight groups (I–VII) based on a phylogenetic tree that also included proteins from two additional *Thalassiosira* species. Chitinases in each group displayed similar domain architectures, motif compositions (Fig. 2), and conserved secondary (Fig. 5) and 3D structure (Table S6), suggesting that they may have a closer evolutionary history and similar cellular functions.

Over the course of evolution, segmental and tandem duplications have propelled the expansion of gene families [36]. Here, we found two tandemly duplicated pairs of *T. pseudonana* chitinase genes (*TpChi11* and *TpChi12*, *TpChi21* and *TpChi22*) and no segmental duplication events, showing that tandem duplication has played a more important role in the expansion of the *T. pseudonana* chitinase gene family. *TpChi21* and *TpChi22* were located close together on Chr19a_19; they were classified into GroupIII (Figs. 2 and 3), and exhibited conserved domains and motif compositions (Fig. 1), as well as similar secondary and 3D structures (Fig. 5 and Table S6). The other pair, *TpChi11* and *TpChi12*, were distantly located on Chr_7 (362 kb) and were classified into different phylogenetic groups. Chitinases TpChi11 and TpChi12 contained a common Glyco_hydro_18 domain. However, TpChi12 also possessed a Glyco_hydro_19 domain and a longer sequence length. Genetic and functional divergence of duplicated genes are known to occur during evolution [37]. *TpChi12* was identified as a tandem duplicate of *TpChi11*, although it had an additional chitin-degrading domain. These observations suggest either the acquisition of a Glyco_hydro_19 domain by *TpChi12* or its loss in *TpChi11*. Furthermore, the exon-intron structures and the gene expression profiles under silica limitation differed between *TpChi11* and *TpChi12*, indicating that this gene pair has undergone functional divergence. Based on these results, we speculate that the chitinase gene family may have undergone duplication first, then experienced genetic diversification that drove further functional diversification.

### Several subcellular locations are predicted for the *TpChi* proteins

Chitin fibrils are located between the silica frustule and the cell membrane of chitin-producing diatoms [38], but the locations of chitin degradation sites are unknown. We therefore investigated the subcellular locations of the *T. pseudonana* chitinases. We mainly interpreted the subcelluar locations of the *TpChi* proteins according to the results of HECTAR that is professionally designed for heterokonts. HECTAR predictions indicated that half of the chitinase family members are involved in the secretory pathway, consistent with the finding that a majority of chitinase genes in *B. rapa* and *Arabidopsis thaliana* are secretory [24, 27]. This suggests that chitinases have extracellular activity and important in vitro applications.

The secretory chitinases are able to degrade the chitin fibre on the diatom cell walls, and further alter cell biosilica deposition and buoyancy [11, 12]. Moreover, in higher plants, secretory chitinases are a group of pathogenesis-related (PR) proteins that in response to pathogens and abiotic stresses [39–41]. In *indica* rice, transgenic rice lines and their progenies overexpressing a bitter melon chitinase gene showed enhanced resistance to major fungal pathogens of rice [40]. In winter rye, dual function-ing antifreeze proteins were observed to be homologous to apoplastic chitinases [41]. Accordingly, we can speculate that the secretory *T. pseudonana* chitinases were involved in the response to chitin-constituting pathogens and abiotic factors, which were also supported by the qRT-PCR results. The remaining chitinases, however, were predicted to reside on the chloroplast or mitochondrial membranes. Given the secondary endosymbiosis exhibited by diatoms [42], *T. pseudonana* chitinases anchored to the chloroplast or mitochondrial membranes may be encoded by genes originally acquired from red algae or non-photosynthetic exosymbionts.

### Different chitinase gene expression patterns may support chitin metabolism and other physiological needs of *T. pseudonana* in changeable environments

Twenty-six types of stress-related *cis*-regulatory elements were found in the promoter regions of the 24 *TpChi* genes (Fig. 4), indicating that they may undergo complex transcriptional regulation. A total of 199 light-responsive *cis-*acting elements were unevenly distributed in the *TpChi* gene promoters, suggesting that the expression of most chitinase genes was indispensably induced by light. Consistent with this observation, LTR, TC-rich repeat, and MBS elements are also important for the induction of chitinase genes in cucumber, *B. rapa, B. juncea*, and *Camelina sativa* [27, 28, 31].

Multiple studies have demonstrated variations in the transcription of chitinase family genes during biotic disease resistance in plants [27, 28, 31]. Cao & Tan (2019) investigated the expression of tomato chitinase genes in response to the pathogen *Sclerotinia sclerotiorum* and under low temperature, high temperature, drought, and salt stresses [29]. They observed that the expression of chitinase genes was broadly elevated under these stress conditions. The present study demonstrated a similar pattern of overall upregulation of *TpChi* genes in response to low irradiance, low temperature, and silica depletion. The different transcriptional profiles of *T. pseudonana* chitinase genes under different abiotic stress conditions suggest that they have various response and regulation mechanisms and may be involved in a variety of physiological processes.

No significant change in in vivo chitinase activity was detected in response to abiotic stresses, despite the increase in chitinase gene transcripts. This can probably be ascribed to the restriction in enzymatic activity or a decrease in the translation of chitinase proteins in adverse environments. The inconsistency between gene transcription and enzyme activity may support the proposal that *T. pseudonana* chitinase genes are involved in other cellular processes apart from chitin metabolism, or it may represent a compensatory adjustment of chitin metabolism in shifting environments. The inconsistent results also suggest the possibility of post-transcriptional regulation during chitin degradation in *T. pseudonana.* By contrast, the expression of seabuckthorn chitinase genes was elevated at both the transcriptional [43, 44] and protein activity levels [43, 44] under cold stress. Moreover, chitinase activity of cucumber and tobacco also increased after treatment with biotic or abiotic stress inducers [45]. The results presented here suggest that there may be functional differences in the regulation and activity of chitinases in diatoms compared to those of higher plants.

Durkin et al. (2009) discovered that silica deposition and chitin exposure on the *T. pseudonana* cell wall were inversely correlated [33]. They suggested that chitin biosynthesis is enhanced in stressed cells that are unable to precipitate silica. In addition, co-regulation of transcript quantity by silicic acid and iron depletion is reported to be associated with cell wall processes [46]. In the present study, chitinase transcripts accumulated while enzymatic activity remained steady under silica depletion. Therefore, stable chitinase activity and higher chitin accumulation may allow chitin to serve as a substitute cell wall material when silica cannot be deposited. Moreover, as silica is required for diatom cell growth, chitin metabolism may also be affected by the abnormal growth of cells, further affecting cycles of carbon and other nutrients in the ocean [47]. However, further investigation of other genes involved in chitin metabolism (e.g., chitin synthases and chitin deacetylases) is required.

### Auxiliary domains may assist in chitin binding by the *T. pseudonana* chitinases

Most chitinases contain a catalytic domain and additional auxiliary domains such as carbohydrate-binding modules (CBMs) [48], which are classified into 87 families at the CAZy database (http://www.cazy.org/). The CBMs anchor the enzyme to the substrate and disrupt its crystalline structure, creating free chain ends and thereby enhancing the enzyme’s catalytic activity towards insoluble substrates [48, 49]. Chitin binding has been demonstrated for a protein containing only two CBM_14 domains [50]. In the present study, the CBM_14, Chitin_bind_1, and LMPO_10 domains, which are typically found in chitin-binding proteins, were identified in the protein sequences of chitinase TpChi3*,* TpChi5*,* TpChi8*,* TpChi11*,* TpChi16*,* TpChi17*,* TpChi21*,* TpChi22*,* TpChi23, and TpChi24 (41.7%), five of which possessed two or more CBM_14 domains (TpChi3*,* TpChi21*,* TpChi22*,* TpChi23*,* and TpChi24) (Fig. 1), suggesting chitin-binding ability of *T. pseudonana* chitinases. Moreover, seven chitinases (TpChi3, TpChi11, TpChi16, TpChi21, TpChi22, TpChi23, and TpChi24) that contained the CBM_14 domain were predicted to be extracellular (containing a signal peptide) or located in the chloroplast or mitochondrial membrane (Table S1). This finding suggests more subcellular location of diatom chitinases containing the CBM_14 domain than the space between the plasma membrane and the silica frustule of diatoms as Traller et al. (2016) discussed [17].

## Conclusions

We identified 24 *TpChi* genes from the *T. pseudonana* genome and divided them into eight subgroups. The gene family was analyzed from the perspective of gene structure, phylogeny, scaffold location, gene duplication, secondary and 3D structures, and expression at the transcriptional and enzymatic levels under abiotic stresses. In general, chitinase transcript levels were upregulated but enzymatic activity remained unchanged under abiotic stresses. These findings suggest that the *T. pseudonana* chitinase gene family is crucial for responses to changeable environments and plays an indispensable as yet uncharacterized role in cellular processes. Our report contributes to a better understanding of chitin metabolism in diatoms and paves the way for future functional characterization of the *TpChi* genes and the in vitro application of *T. pseudonana* chitinases.

## Methods

### *T. pseudonana* cultivation

*T. pseudonana* (CCMP 1335) was obtained from the Microalgae Collection Center at Ningbo University (Ningbo, Zhejiang, China). Cells were grown in optimized f/2 liquid medium supplied by Shanghai Guangyu Biological Technology Co., Ltd. Cultures were grown at 19 °C with a 12 h: 12 h light: dark diurnal cycle (100 μmol m^− 2^ s^− 1^) and shaken at 100 rpm.

When cells grown in the starter culture had reached the exponential phase, 50 mL of cell culture were centrifuged under sterile conditions at 2850 *g* for 15 min (Beckman Coulter, Allegra X-I5R Centrifuge), washed once with medium, and inoculated into 50 mL of fresh medium. For temperature-limited treatment, cells were placed under a low temperature of 12 °C with 100 μmol m^− 2^ s^− 1^ of light, whereas for the light-limited treatment, cells were exposed to a low irradiance of 50 μmol m^− 2^ s^− 1^ at 19 °C. For the silica-limited treatment, cells were washed once with medium and inoculated into 50 mL of fresh, silica-free medium and were then cultured under normal light (100 μmol m^− 2^ s^− 1^) and temperature (19 °C) conditions with the control group. The experiment was performed in flasks for 48 h, and there were three biological replicates per treatment.

### Identification of chitinase family genes in *T. pseudonana*

The nucleotide sequences and corresponding protein sequences of *T. pseudonana* chitinase genes were retrieved from the Joint Genomics Institute PhycoCosm database (JGI PhycoCosm, https://jgi.doe.gov/data-and-tools/phycocosm/) using “chitinase” and other relevant keywords listed in Table S7. A total of 93 hits were annotated as chitinase-relevant genes. From these, we removed 41 genes that were redundantly annotated as full-length sequences but were in fact partial sequences of the other 52 genes (Table S8). Protein sequences of the remaining 52 genes were submitted to the SMART and Pfam websites to confirm the presence of chitinase domains Glyco_hydro_18 (PF00704) and Glyco_hydro_19 (PF00182) with a cut-off *E*-value of < 0.0001 [51–53]. Proteins with one or both of the Glyco_hydro_18 and Glyco_hydro_19 domains were regarded as putative chitinases.

### Sequence analysis and structural characterization

All of the nucleotide sequences were analyzed using the ExPASy ProtParam tool to calculate their amino acid numbers, molecular weights, andpIs [54]. Genomic locations, intron numbers, and gene structure information were acquired from JGI PhycoCosm. MEME V5.1.1 online software was used to identify conserved motifs in the chitinase proteins with the following parameters: any number of repetitions, maximum of 20 motifs, and an optimum motif width of 6–200 amino acids [55]. Signal peptides and TMHs were predicted using SignalP-5.0 (http://www.cbs.dtu.dk/services/SignalP/) and TMHMM Server v.2.0 (http://www.cbs.dtu.dk/services/TMHMM-2.0/), respectively. Subcellular locations were predicted by combining the results of WoLF PSORT (https://wolfpsort.hgc.jp/) with those of HECTAR and ASAFind, the two software that are specific for heterokonts [56–58].

Secondary structures of *T. pseudonana* chitinase protein sequences were predicted using SOPMA (https://npsa-prabi.ibcp.fr/cgi-bin/npsa_automat.pl?page=/NPSA/npsa_sopma.html) with default parameters. For each phylogenetic group of *T. pseudonana* chitinases that contained at least two members, protein sequences were aligned with ClustalW (https://www.genome.jp/tools-bin/clustalw), and representative secondary structures were then visualized with ESPript3.0 [59]. 3D models of the *T. pseudonana* chitinases were constructed using the SWISS-MODEL server [60–64].

### Scaffold localization and gene duplication identification

TBtools software was used to perform scaffold localization based on retrieved genomic location information [65]. Gene duplication events within the family were identified with BLASTp and MCScanX methods [66]. All the *T. pseudonana* chitinase protein sequences were aligned with each other using BLASTp. The BLASTp result was used as input for MCScanX, and duplication events and gene collinearity were identified using default parameters.

### Phylogenetic analysis and classification

To study the evolutionary relationships among chitinases from *T. pseudonana*, *Thalassiosira oceanica,* and *Thalassiosira weissflogii*, an unrooted neighbor-joining phylogenetic tree was constructed using MEGA X software (v10.1.8) with 1000 bootstrap replicates [67]. The *T. oceanica* protein sequences were identified and downloaded from the JGI PhycoCosm database using the keyword “chitinase”. To acquire *T. weissflogii* protein sequences, the 24 *T. pseudonana* chitinase protein sequences were used as BLASTp queries against the *T. weissflogii* transcriptomes released by the Marine Microbial Eukaryote Transcriptome Sequencing Project (MMETSP) [68]. The *T. oceanica* and *T. weissflogii* candidate sequences were submitted to SMART and Pfam to verify the presence of one or both of the Glyco_hydro_18 and Glyco_hydro_19 domains [51–53]. In total, 22 full-length *T. oceanica* chitinases (Table S3), 76 *T. weissflogii* chitinases (Table S4), and the 24 newly identified chitinases from *T. pseudonana* were used for phylogenetic analysis.

### Prediction of *cis*-acting elements

To predict the *cis-*acting regulatory elements in the promoter regions of *T. pseudonana* chitinase genes, 1.5 kb of sequence upstream from each initial codon (ATG) was downloaded from the JGI PhycoCosm database. Stress response-related *cis-*acting elements in the promoter sequences of the chitinase genes were then investigated using the PlantCARE website (http://bioinformatics.psb.ugent.be/webtools/plantcare/html/) [69].

### Enzyme assays and transcriptional profile analysis

After 48 h of stress treatment, a 1 mL sample was taken from each flask for measurement of chitinase activity using the Solarbio Chitinase Assay Kit (Cat#BC0820) following the manufacturer’s instructions. The remaining 49 mL of *T. pseudonana* cells in each flask were collected by centrifugation at 2850 *g*, washed once with fresh medium, and flash-frozen in liquid nitrogen. The *T. pseudonana* cell pellets were homogenized by vortexing in TRIzol reagent (Invitrogen, Waltham, MA, USA), then centrifuged at 9700 *g*. The supernatants were used for RNA extraction with chloroform and isopropanol. RNA pellets were resuspended in diethyl pyrocarbonate-treated water, followed by elimination of genomic DNA and synthesis of complementary DNA (cDNA) with a PrimeScript RT reagent kit (Takara, Japan). The synthesized cDNA was then used for qRT–PCR experiments.

The transcriptional profiles of 14 randomly selected *T. pseudonana* chitinase genes were obtained by qRT–PCR using TB Green™ Premix Ex Taq™ II (Takara, Japan) and a Thermal Cycle Dice™ Real Time System (Takara, Japan). Gene-specific qRT–PCR primers were designed using the NCBI Primer-BLAST website [70]. Information on primers is provided in Table S9. The beta tubulin gene (TUB3) was used as the reference gene for normalizing the expression of the *T. pseudonana* chitinase genes [71].

## Supplementary Information

**Additional file 1: Table S1.** Analysis and prediction of signal peptides, Transmembrane helices (TMHs) and Subcellular localizations of the TpChi proteins.

**Additional file 2: Table S2.** List of the putative motifs of the TpChi proteins.

**Additional file 3: Table S3.** The information of chitinases from *T. oceanica*.

**Additional file 4: Table S4.** The information of chitinase from *T. weissflogii*.

**Additional file 5: Table S5.** The proportion of secondary structure of the TpChi proteins.

**Additional file 6: Table S6.** Top 3D models predicted of of the TpChi proteins.

**Additional file 7: Table S7.** Keywords used for searching T. pseudonana chitinase genes on JGI.

**Additional file 8: Table S8.** All transcripts annotated as chitinase in T. pseudonana genome from JGI database.

**Additional file 9: Table S9.** Primers used in qRT-PCR studies.

## Data Availability

The reference genome of *T. pseudonana* was released in JGI PhycoCosm with the accession number of *Thalassiosira pseudonana* CCMP 1335 (https://jgi.doe.gov/data-and-tools/phycocosm/). Gene sequences and protein sequences of *T. pseudonana* chitinase were downloaded from JGI PhycoCosm (https://jgi.doe.gov/data-and-tools/phycocosm/). Chitinase protein sequences of *T. oceanica* were downloaded from JGI PhycoCosm (https://jgi.doe.gov/data-and-tools/phycocosm/). Chitinase protein sequences of *T. weissflogii* were downloaded from iMicrobe website (https://www.imicrobe.us/). All of the datasets supporting the results of this article are included within the article and its additional files.

## Abbreviations

AA: Amino acid
AS: Alternative splicing
CBMs: Carbohydrate-binding modules
JGI PhycoCosm: Joint Genomics Institute PhycoCosm database
MW: Molecular weight
NCBI: National Center for Biotechnology Information
NJ: Neighbor-joining
ORFs: Open reading frames
pI: Isoelectric point
qRT–PCR: Quantitative real-time polymerase chain reaction
TMHs: Transmembrane helices
UTR: Untranslated region
3D: Three dimensional
